# Potential of a Composite Conduit with Bacterial Nanocellulose and Fish Gelatin for Application as Small-Diameter Artificial Blood Vessel

**DOI:** 10.3390/polym14204367

**Published:** 2022-10-17

**Authors:** Luhan Bao, Can Li, Man Tang, Lin Chen, Feng F. Hong

**Affiliations:** 1 State Key Laboratory of Biobased Material and Green Papermaking, Qilu University of Technology, Shandong Academy of Sciences, Jinan 250353, China; 2 Group of Microbiological Engineering and Biomedical Materials, College of Biological Science and Medical Engineering, Donghua University, North Ren Min Road 2999, Shanghai 201620, China; 3 Scientific Research Base of Bacterial Nanofiber Manufacturing and Composite Technology, China Textile Engineering Society, Shanghai 201620, China

**Keywords:** bacterial nanocellulose, fish gelatin, small-diameter artificial blood vessel, composite conduit

## Abstract

Bacterial nanocellulose (BNC) has received great attention for application as an artificial blood vessel material. However, many results showed that pristine BNC could not perfectly meet all the demands of blood vessels, especially for rapid endothelialization. In order to improve the properties of small-caliber vessels, different concentrations of fish gelatin (Gel) were deposited into the 3D network tubes and their properties were explored. The BNC/Gel composite tubes were treated with glutaraldehyde to crosslink BNC and fish gelatin. Compared with pristine BNC tubes, the BNC/Gel tubes had a certain improvement in mechanical properties. In vitro cell culture demonstrated that the human endothelial cells (HUVECs) and human smooth muscle cells (HSMCs) planted on the internal walls of BNC/Gel tubes showed better adhesion, higher proliferation and differentiation potential, and a better anticoagulation property, as compared to the cells cultured on pristine BNC tubes. Whole-blood coagulation experiments showed that the BNC/Gel tube had better properties than the BNC tube, and the hemolysis rate of all samples was less than 1.0%, satisfying the international standards for medical materials. An increase in the content of fish gelatin also increased the mechanical properties and the biocompatibility of small-caliber vessels. Considering the properties of BNC/Gel tubes, 1.0 wt/v% was selected as the most appropriate concentration of fish gelatin for a composite.

## 1. Introduction

Cardiovascular diseases are one of the leading causes of mortality and morbidity worldwide [1]. Each year, cardiovascular diseases cause more than 4.3 million deaths in Europe [2]. Vessel transplantation is the major treatment for vascular disease, which is a common disease posing a great threat to patients’ lives [3]. Vascular grafts are generally divided into three types: autologous, allogeneic and synthetic blood vessels. Among them, autologous blood vessels are most suitable. However, the limited source of autologous blood vessels cannot meet the large number of clinical demands [4]. Some types of materials, such as expanded polytetra fluoroethy (ePTFE) and polyethylene terephthalate (PET), are commercially available and clinically applied for medium- and large-caliber artificial blood vessels (inner diameter ≥ 6 mm) [5]. However, some researchers have shown that ePTFE and PET have poor compliance and a low patency rate as applied for small-caliber (inner diameter < 6 mm) artificial blood vessels [6]. Therefore, it has become necessary to develop and investigate suitable materials for application as small-caliber artificial blood vessels.

The ideal artificial blood vessel should have the following characteristics: sufficient strength, excellent compliance and resistance, long patency, great histocompatibility and hemocompatibility [1,7]. Bacterial nanocellulose (BNC), also known as bacterial cellulose (BC), is a unique material with promising biocompatibility, high water-holding capacity, good mechanical properties, a 3D nanofibril network structure (an extracellular matrix-like structure) and high crystallinity and purity [8]; it has been considered as a promising artificial blood vessel material. At present, different BNC tubes harvested from different types of bioreactors and their great potential for artificial blood vessels have been reported [9,10,11]. Moreover, the excellent porous structure can support the adhesion and high proliferation of cells [2,9]. Some animal experiments have also shown that BNC tubes do not produce a significant inflammatory reaction [8]. However, some properties of BNC tubes do not satisfactorily achieve the demands of clinical application as artificial blood vessels, such as mechanical properties in a hydrogel state, anticoagulation biocompatibility and other characteristics [12,13]. Therefore, BNC must be modified to better satisfy these requirements as an artificial blood vessel material.

BNC belongs to the group of polysaccharides, with low bioactivity and chemical activity, while the collagen hydrogel belongs to the group of proteins, with cell surface receptors [14]. Gelatin, as a degradation product of collagen, inherits the unique properties of collagen, such as biocompatibility, processability, biodegradability and the promotion of cell differentiation and proliferation. Additionally, compared to collagen, gelatin molecules have more active side groups exposed, and the chemical reaction activity is higher, which is favorable for chemical modification [15]. Since the production cost of gelatin is cheaper, it has great advantages in actual production and application, especially in drug delivery [16]. It has been reported that in a composite of BNC and gelatin, BNC will act as a scaffold and gelatin will fill in the BNC network. Hydrogen bonds are the major conjugate force between gelatin and cellulose [17], and the composite affects cells’ proliferation in a positive way with the growth factor of gelatin [18]. Fish gelatin was chosen in this study because it does not pose a restriction regarding religion and health [19], satisfying the needs of all people. Additionally, due to the concerns regarding bovine diseases, especially the risk of bovine spongiform encephalopathy (BSE), foot and mouth disease arising from the cow and pig, the use of bovine gelatin is limited [20]. Gelatin derived from different animals and tissues has different components and properties. The amino acid composition of fish-derived gelatin is broader than that from terrestrial mammals [20]. It is reported that grass carp skin gelatin has better compatibility with gelatin from bovine and porcine skins [21]. The development of fish skin gelatin has therefore attracted researchers’ attention.

In order to construct small-caliber artificial blood vessels with better bioactivity and promotion of endothelialization, fish-derived gelatin is therefore considered for compounding with BNC. To the best of our knowledge, BNC/Gelatin (BNC/Gel) artificial blood vessels have not been reported. In this study, it was anticipated that the synthesis of BNC/Gel composite tubes may endow the BNC matrix with anticoagulant properties and prompt the proliferation of human endothelial and smooth muscle cells, both of which are the main cell types in natural blood vessels. The typical nature of a blood vessel is that it is composed of three layers. The inner layer consists of vascular endothelial cells (VECs), the middle layer consists of smooth muscle, and the outer layer consists of connective fibers [22]. The BNC tubes were prepared via in situ cultivation in a patented bioreactor device, G-BNC [8], and glutaraldehyde was used as a chemical crosslinker after the introduction of fish gelatin into the fiber network of BNC tubes. The study also explored the mechanical properties, anticoagulant properties and biocompatibility of the BNC/Gel composite tubes obtained with four different concentrations of fish gelatin. The performance of BNC/Gel composite tubes was compared with that of pristine BNC tubes for application as small-caliber artificial blood vessels by evaluating the physicochemical properties, hemocompatibility and cytocompatibility. This is the first study to explore the combination of different concentrations of fish gelatin with BNC tubes, and demonstrate the potential of the composite tubes in application as small-caliber artificial blood vessels. In addition to fish-derived gelatin, the compositing strategy proposed in the study could lay a foundation for the development of other BNC-based composites.

## 2. Experimental

### 2.1. Materials

BNC producing bacteria strain *Komagataeibacter xylinus* DHU-ATCC-1 was isolated from *K. xylinus* ATCC 23,770 (American Type Culture Collection, Manassas, VA, USA) after routine mutation with UV radiation. Fructose, tryptone, yeast extract powder, citric acid, fish-derived gelatin (from cold water fish skin, Sigma G7041, High Purity Grade) and glutaraldehyde (25% *w/v*%) were purchased from Sinopharm Chemical (Shanghai). Dulbecco’s Modified Eagle’s Medium (DMEM), fetal bovine serum (FBS), Cell Counting Kit-8 (CCK-8), trypsin and penicillin–streptomycin antibody (100×, 10,000 U/mL penicillin and 10,000 μg/mL streptomycin) (Gibco, CA, USA) were supplied by Shanghai Yuanxiang Medical Equipment Co., Ltd. (Shanghai, China). Coomassie Brilliant Blue G250 and FITC Phalloidin were purchased from Shanghai YEASEN Biotechnology Co., Ltd. (Shanghai, China). DAPI was purchased from BestBio Science Co., Ltd. (Shanghai, China). The cell lines of human arterial smooth muscle cells (HSMCs) and human umbilical venous endothelial cells (HUVECs) were purchased from the Institute of Biochemistry and Cell Biology (The Chinese Academy of Sciences, Shanghai, China). The ultra-pure and deionized water was obtained by using a Pall Water Filterpure system (Cascada I, Pall Filter (Beijing) Co., Ltd., Beijing, China).

### 2.2. Production of BNC Tubes

**Figure 1:**
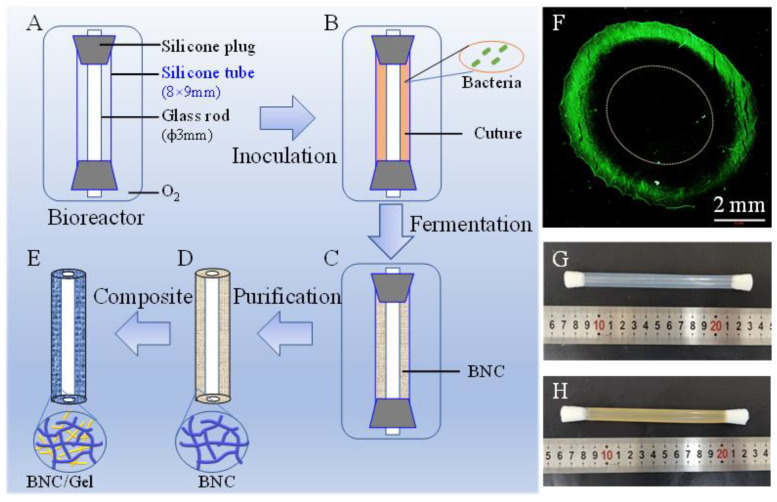
Schematic diagram of production of BNC tube by fermentation, followed by synthesis of BNC/Gel tube. (**A**) The patented device for producing BNC tube; (**B**) inoculation of fermentation broth containing activated bacterial cells; (**C**) formation of BNC tubes in the bioreactor by fermentation; (**D**) BNC tube after purification and its network structure; (**E**) BNC/Gel tube obtained by crosslinking BNC tube and gelatin to form double network structure; (**F**) the distribution of *K. xylinus* cells in the bioreactor, inspected by using nucleic acid dye staining; (**G**) appearance of the bioreactor; (**H**) a BNC tube formed in the bioreactor.

The bioreactor device was assembled with a silicone tube (inner diameter × external diameter: 8 mm × 9 mm, 120 mm in length) and a glass tube (3 mm in diameter) (Figure 1A, Chinese patent 201810585866.1 [9,23]). Seed medium was prepared with 100 g/L fructose, 5 g/L tryptone and 3 g/L yeast extract powder and adjusted pH to 5.0–5.2 with citric acid. After autoclaving for 30 min at 115 °C, the bioreactor and seed medium were placed in a sterile bench and cooled for further use. The strain, kept in a glycerol cryogenic vial, was carefully transferred to the seed medium, and it was shaken at 30 °C for one day. Then, the culture medium was inoculated into the autoclaved bioreactor (Figure 1B). The bioreactor was incubated at 30 °C for 7 days and the oxygen was changed every other day. After incubation, the formed BNC tubes (Figure 1C,D, 100 mm in length) were soaked in a 1% (*w/v*) sodium hydroxide aqueous solution at 80 °C for 4 h to remove medium residue and bacterial cells, and then washed with ultra-pure water repeatedly until neutral. Finally, the BNC tubes were autoclaved more than 5 times and continuously washed with ultra-pure water until endotoxin was less than 0.25 EU/mL.

### 2.3. Preparation of the BNC/Gel Composite Tubes

BNC has pores of approximately 2 μm in the fiber network, which may permit fish gelatin molecules to penetrate into the fibril structure of the BNC tube wall. The pristine BNC tubes (3 mm in inner diameter, cut to 50 mm in length) were soaked in fish gelatin solution at various concentrations (0.25%, 0.5%, 1%, 2%, *w/v*) and shaken at 160 rpm and 30 °C for 24 h. Afterwards, the samples were taken out and thoroughly immersed in 1% (*v/v*) glutaraldehyde solution for 6 h and rinsed with distilled water until the residual glutaraldehyde was washed away to obtain BNC/Gel composite tubes (BNC/Gel-0.25, BNC/Gel-0.5, BNC/Gel-1.0, BNC/Gel-2.0). According to a previous study, the BNC tubes were immersed in 1.0% (*v/v*) glutaraldehyde, which is a well-known and popularly used chemical reagent for crosslinking amino and hydroxyl groups, and it can ensure the good adhesion of gelatin molecules to the fibers of BNC [24,25,26]. In addition, it was reported that 5.0% (*v/v*) glutaraldehyde did not damage the cytocompatibility of a crosslinked material [27]. However, studies have shown that high-concentration glutaraldehyde induces cytotoxicity [28]. Therefore, the best glutaraldehyde concentration for chemical crosslinking is 1.0% (*v/v*), which maintains the cytocompatibility of composites [24,29,30].

### 2.4. Analysis of Fish Gelatin

In order to qualitatively measure the distribution of fish gelatin in the BNC/Gel composite tubes, the pristine BNC and BNC/Gel composite tubes of approximately 30 mm in length were immersed in Coomassie Brilliant Blue G250 solution and shaken at 160 rpm and 30 °C for 24 h. Finally, all samples were rinsed repeatedly with deionized water until the free dye in the tube wall was removed. Images (external and sectional views) of all samples were taken by using a fixed focus camera.

For the determination of the fish gelatin content of BNC/Gel composite tubes, the tubes were precisely cut to 5 cm in length and dried at 80 °C for 24 h, and the weights of the samples were measured. The fish gelatin content of the composite tubes was calculated as follows:

(1) $$WG(\%)=\frac{W_{2}-W_{1}}{W_{2}}\times 100\%$$

where W_1_ and W_2_ were the dry weight of the BNC tube and BNC/Gel composite tube, respectively. All samples were analyzed in triplicate.

### 2.5. Fourier Transform Infrared Spectroscopy (FTIR)

The composition and structure of freeze-dried samples were characterized by using a Perkin Elmer Spectrum-Two (USA) equipped with an ATR accessory in the range of 4000−400 cm^−1^ and at a resolution of 4 cm^−1^.

### 2.6. Morphology Analysis

The microstructures of samples were observed by using a scanning electron microscope (SEM, Phenom XL, Phenom Scientific, Shanghai, China). Samples were freeze-dried; then, samples were attached to the sample table with a conductive adhesive and we held the inner surface upward, and samples were gold-coated before SEM analysis. One hundred fibers were selected randomly on SEM images to measure fiber diameters via image analysis software (Image-J, National Institutes of Health, Bethesda, MD, USA) and for the analysis of the fiber distribution by Origin 8 (OriginLab, Northampton, MA, USA).

### 2.7. Physical and Mechanical Properties

The water permeability and burst pressure of tubes were measured in accordance with methods that have been described previously [10]. They were measured with a home-made instrument consisting of a pressure gauge and a syringe. For water permeability measurement, the intraluminal pressure of the tube was maintained at 0.016 MPa within one minute, and the amount of water passing through the tube wall was recorded and calculated in mL cm^−2^ min^−1^. For burst pressure measurement, water was injected into the tubes (40 mm in length), and the pressure was increased by 0.02 MPa every 10 s. The burst pressure at the point of the sudden breakage of the tube was recorded. All samples were analyzed in quadruplicate.

Tensile strength was tested by using a universal material testing machine (H5K-S, Tinius Olsen, Philadelphia, PA, USA) at a stretching speed of 50 mm min^−1^ at room temperature. The mean value was obtained from at least four tubular samples of 5 cm in length.

### 2.8. Hemocompatibility Tests

Hemocompatibility tests including hemolytic rate, plasma recalcification profile and whole blood clotting time measurements were performed in accordance with the methods described in detail in our previous study [25]. Whole blood was collected from a rabbit auricular vein by using 5 mL vacuum blood collection tubes containing 3.8% (*w/v*) citrate as an anticoagulant. The experimental procedures relating to rabbits were approved by the Laboratory Animal Ethics Review Committee of Donghua University (DHUEC-NSFC-2019-13) and were conducted in accordance with ethical norms, using protocols consistent with the Guide for the Care and Use of Laboratory Animals (Ministry of Science and Technology of China, 2006). Platelet-Rich Plasma (PRP) was obtained by centrifugation at 100× *g* and 4 °C for 10 min, which was then used in the platelet adhesion assay. The red blood cells at the bottom of the centrifuge tube were harvested to be used for hemolytic ratio measurement after increasing the volume to 5 mL with normal saline. In addition, Platelet-Poor Plasma (PPP) was obtained by centrifugation at 560× *g* and 4 °C for 10 min, which was then used in the plasma recalcification profile assay. In the assays, sterilized samples were cut into 1 cm × 1 cm sections and tiled at the bottom of a 24-well plate with a sufficient amount of normal saline.

(1) Hemolytic rate

The hemolytic ratio depended on the quantity of hemoglobin released by lysed red blood cells. The samples were soaked in 2 mL normal saline, along with empty polystyrene plate wells containing 2 mL ultra-pure water (positive control) and plate wells containing 2 mL normal saline (negative control), and were incubated at 37 °C for 1 h. Approximately 1.5 × 10^8^ red blood cells were added into each well, followed by further incubation at 37 °C for 1 h. Then, we took 1 mL solution from each well and centrifuged it at 2500 rpm for 5 min. After this, the absorbance of 100 μL supernatant at 540 nm was measured by transferring it into a 96-well plate using a microplate reader (Multiskan MK3, Thermo Labsystem, Waltham, MA, USA). Each sample was measured in triplicate. The hemolytic rate was calculated as follows: hemolytic ratio (%) = (OD_test_ − OD_neg_)/(OD_pos_ − OD_neg_) × 100%, where OD_test_, OD_neg_ and OD_pos_ are the absorbance of the test sample, negative control and positive control, respectively.

(2) Plasma recalcification profile

Samples along with blank plate wells were incubated with 500 μL PRP at 37 °C for 2 h. After this, an amount of 100 μL from each sample well (control groups from blank plate well) was transferred to a 96-well plate and 100 μL 0.025 M CaCl_2_ was added immediately, with the exception of the negative control (100 μL 0.025 M CaCl_2_ for positive control and 100 μL normal saline for the negative control). Then, the 96-well plate was placed in a microplate reader and the absorbance at 405 nm was recorded every 30 s for 45 min. Each sample was measured in triplicate and the mean value was calculated.

(3) Whole blood clotting time

Whole blood clotting times on BNC and its composite tubes were measured as previously described [25]. Firstly, CaCl_2_ (0.1 M) of 500 μL was added to 5 mL whole blood and mixed well to induce clotting. Then, each sample in the 24-well plate was combined with the above mixture of 100 μL and incubated for 5, 10, 20 and 40 min at 37 °C, respectively. After this, at the end of each time point, ultra-pure water at a volume of 2.5 mL was added into the wells and they were incubated for another 5 min. Finally, the absorbance values of the supernatant were measured at 540 nm, which reflected the released hemoglobin. 

### 2.9. In Vitro Cytocompatibility

For the cytocompatibility assay, the samples were cut into 1 cm × 1 cm sections and placed in 24-well plates with the internal surface facing upward. Then, the samples were sterilized in 75% (*v/v*) ethanol for 12 h, followed by thorough washing with sterile PBS to eliminate ethanol for further use.

The HUVECs (1 × 10^4^) were seeded on the internal surfaces of these samples, with the addition of 400 μL DMEM medium supplemented with 10% (*v/v*) FBS and 1% (*v/v*) penicillin–streptomycin antibody in each well [13]. After incubation for 1, 3, 5 days, the samples were rinsed with PBS 3 times, a mixture of 360 μL DMEM and 40 μL CCK-8 was added into each sample, and they were incubated at 37 °C for 1 h. Then, an aliquot of 100 μL was transferred to a 96-well plate, and the absorbance at 450 nm was measured by using a microplate reader (Multiskan MK3, Thermo Fisher, Shanghai, China). Three parallel replicates were read for each sample. After this, the cultured samples with HUVECs were washed with PBS at each time point (1, 3, 5 days), followed by immobilization with 2 mL 2.5% (*v/v*) glutaraldehyde solution for 4 h. After this, the samples were washed with ultra-pure water and dehydrated in an ascending series of ethanol solutions (25%, 50%, 75%, 95% and 100%) for 15 min each time. After dehydration, tertiary butanol was added to replace the ethanol three times (soaked 15 min for each time) and then the samples were freeze-dried. Finally, the cell morphologies were observed using SEM (Phenom XL, Phenom Scientific, Shanghai, China) after samples were sputtered with gold.

The HSMCs (1 × 10^4^) were seeded on the internal surfaces of these samples. After incubation for 1, 3, 5 days, the CCK-8 test result was consistent with that of HUVECs. FITC-Phalloidin and DAPI were used to stain the cells’ cytoskeleton and nucleus at each time point, and then images were taken using fluorescent microscopy. 

For the further observation of cell morphology, the incubated HUVECs and HSMCs were washed with PBS three times and then fixed with 2 mL 2.5% (*v/v*) glutaraldehyde for 4 h. Then, all samples were washed with PBS three times and dehydrated by adding various concentration gradients of ethanol (25%, 50%, 75%, 95% and 100%), each concentration for 15 min. Ethanol was replaced with t-butanol 3 times for 15 min each time, and then the samples were freeze-dried. Finally, the cell morphologies were observed by using SEM (Phenom XL, Phenom Scientific, Shanghai, China).

## 3. Results and Discussion

### 3.1. Gelatin Distribution Measurement and Morphology Inspection

**Figure 2:**
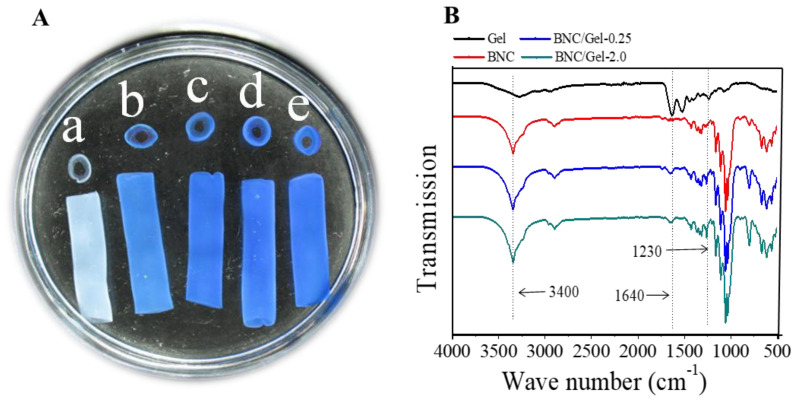
(**A**) Macromorphology with gelatin uniformity visualization for BNC tube (a), BNC/Gel−0.25 (b), BNC/Gel−0.5 (c), BNC/Gel−1.0 (d) and BNC/Gel−2.0 (e) after Coomassie Brilliant Blue G250 staining. (**B**) FTIR spectra of BNC tube and BNC/Gel composite tubes.

In order to measure the distribution of fish gelatin in the BNC/Gel composite tubes, staining with Coomassie Brilliant Blue G250 was used based on the specific binding between the dye and proteins [31]. As shown in Figure 2A, the BNC/Gel composite tubes exhibited a distinctly uniform blue color, and the more fish gelatin included in the composite, the deeper the color; however, BNC/Gel-1.0 and BNC/Gel-2.0 had no obvious color difference. By contrast, the pristine BNC tube was not stained, indicating only the existence of fish gelatin in the BNC/Gel composite tubes. However, the amount of gelatin deposited into the 3D network tubes was difficult to quantify. Therefore, the content of fish gelatin was calculated based on the dry weights of BNC and BNC/Gel composite tubes, as shown in Table 1. The result showed that BNC/Gel-2.0 tubes contained higher fish gelatin content of approximately 0.23%. The results were consistent with the staining results obtained using Coomassie Brilliant Blue G250.

Figure 2B shows the FTIR spectra of fish gelatin, pristine BNC and BNC/Gel tubes. For BNC, the strong band at 3440 cm^−1^ reflected the stretching vibration of the oxygen hydrogen bond (O-H), and 1250–850 cm^−1^ reflected the stretching vibration peak of C-O-C. The peaks around 1230–1240 cm^−1^ were the amide (N-H) vibration and amide III (C-N) stretching vibration peaks of gelatin. For the BNC/Gel composites, the absorption peak of fish gelatin for the BNC/Gel-2.0 tube was sharper than that of BNC, which might be ascribed to the fact that more gelatin molecules were coated on the surfaces of the BNC/Gel-2.0 tubes.

**Figure 3:**
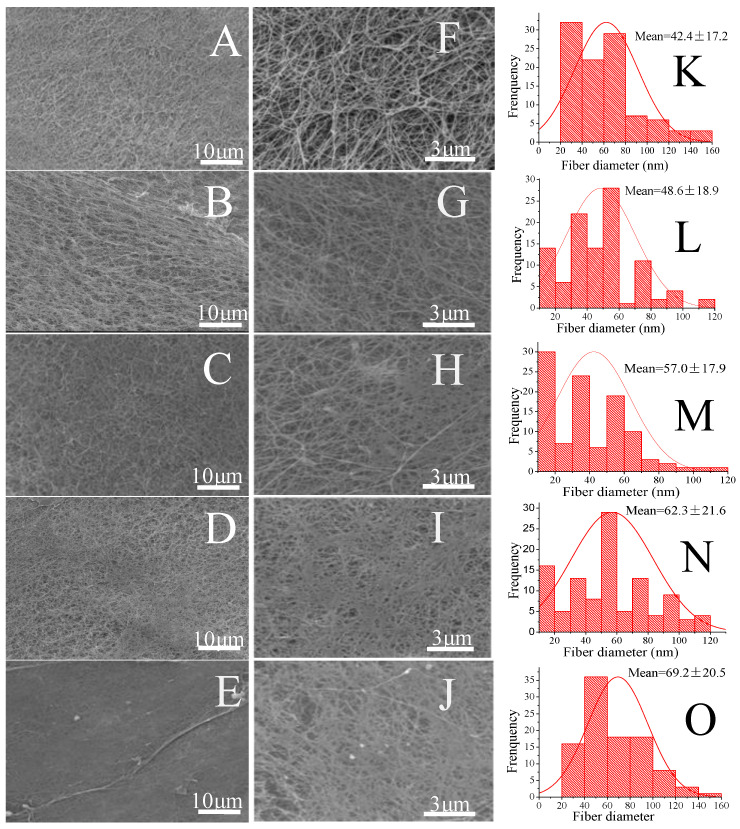
Microstructure and fiber diameter distributions in tube walls of BNC and BNC/Gel composite tubes. SEM images (**A**–**E**) (5000×) and (**F**–**J**) (20,000×) represent the BNC tube, BNC/Gel-0.25, BNC/Gel-0.5 tube, BNC/Gel-1.0 tube and BNC/Gel-2.0 tube. The fiber diameter distribution (**K**–**O**) is the same as the order of (**A**–**F**).

The inner surface microstructures of BNC and BNC/Gel composite tubes can be observed from the SEM results in Figure 3. The results showed that all the tubes maintained a nanofiber network. The mean values of fiber diameter are also shown in Figure 3. The mean diameter of the fibers in the BNC tube wall was smaller than that of the composite tubes, and the mean fiber diameter became larger as the soaked gelatin concentration increased. As shown in Figure 3K–O, the introduction of fish gelatin could increase the average diameter of the fibers (42.4 nm compared to 69.2 nm), and the diameter increased as the amount of gelatin increased. The reason might be the fact that gelatin adhered to and enwrapped the fibers, and some of it entered the fiber gaps to form a denser structure, which all resulted in thicker fibers.

### 3.2. Water Permeability and Mechanical Properties

Water permeability is one of the major evaluation factors for artificial blood vessels and grafts. It reflects the porous structure of the fiber network and the capacity to exchange nutrients and metabolize waste, which may play an important role in cell and tissue growth. Conduits with too high or too low water permeability are not suitable for application as artificial blood vessels, which means that conduits without water permeability are not conducive to the delivery of nutrients and discharge of waste, and conduits with high water permeability would cause blood leakage [32]. As shown in Table 1, the water permeability of different BNC/Gel composite tubes was approximately 4.2, 4.5, 4.6 and 5.2 mL cm^−2^ min^−1^, respectively. As compared with the water permeability of the BNC tube (4.2 mL cm^−2^ min^−1^), the BNC/Gel composite tubes had a better capacity for water and nutrient transportation through the tube walls. A previous study reported that ePTFE possesses water permeability of approximately 0.83 mL cm^−2^ min^−1^, which is much lower than BNC tubes, indicating that nutrients and water are not easily exchanged [8].

The burst pressure of the BNC tube was around 660.1 mmHg (0.088 MPa), and that of the BNC/Gel composite tubes was slightly lower. The explanation for this phenomenon might be ascribed to the fact that the chemical crosslinking with glutaraldehyde led the weakest part of the BNC/Gel composite tubes to become brittle, and the weak points were more likely to break when pressure was applied. However, all BNC/Gel composite tubes can bear pressure three times or higher than the human normal blood pressure (90–140 mmHg), indicating that the burst pressures of all tubes satisfy the requirements of vascular grafts. According to the previous results, ePTFE possesses pressure of more than 1875 mm Hg (0.25 MPa), exceeding the measurement range of the test device. The burst pressures and tensile strengths of BNC conduits in a gel state are not comparable with those of the ePTFE graft as it is in a dry state with a dense network structure [8].

As shown in Table 1, the axial tensile strength of BNC and BNC/Gel composite tubes was also studied, and good mechanical performance was shown. For the BNC tube, the Young’s modulus reached 0.98 MPa, and those of the BNC/Gel composite tubes were higher. The elongation at break of most of the BNC/Gel composite tubes (38.8–43.9%) was also superior to that of the BNC tube (38.6%), indicating that the BNC/Gel composite tubes possessed better mechanical properties.

From comprehensive analysis of BNC’s mechanical reinforcement effect, the main factors are that (i) BNC is a nanofiber material with high crystallinity and high mechanical properties [33]; (ii) the BNC surface contains a large amount of hydroxyl groups, which have strong hydrophilicity and easily form hydrogen bonds with amino groups in fish gelatin molecules [34], further improving the mechanical properties of the composite scaffold; (iii) the fish gelatin molecules may be distributed in the pores of tube walls and inside the gaps of the BNC nanofibers to form a three-dimensional nano-network structure, which is beneficial for the improvement of the mechanical properties [35].

### 3.3. Evaluation of Blood Compatibility of Composite Tubes

(1) Hemolytic ratio

**Figure 4:**
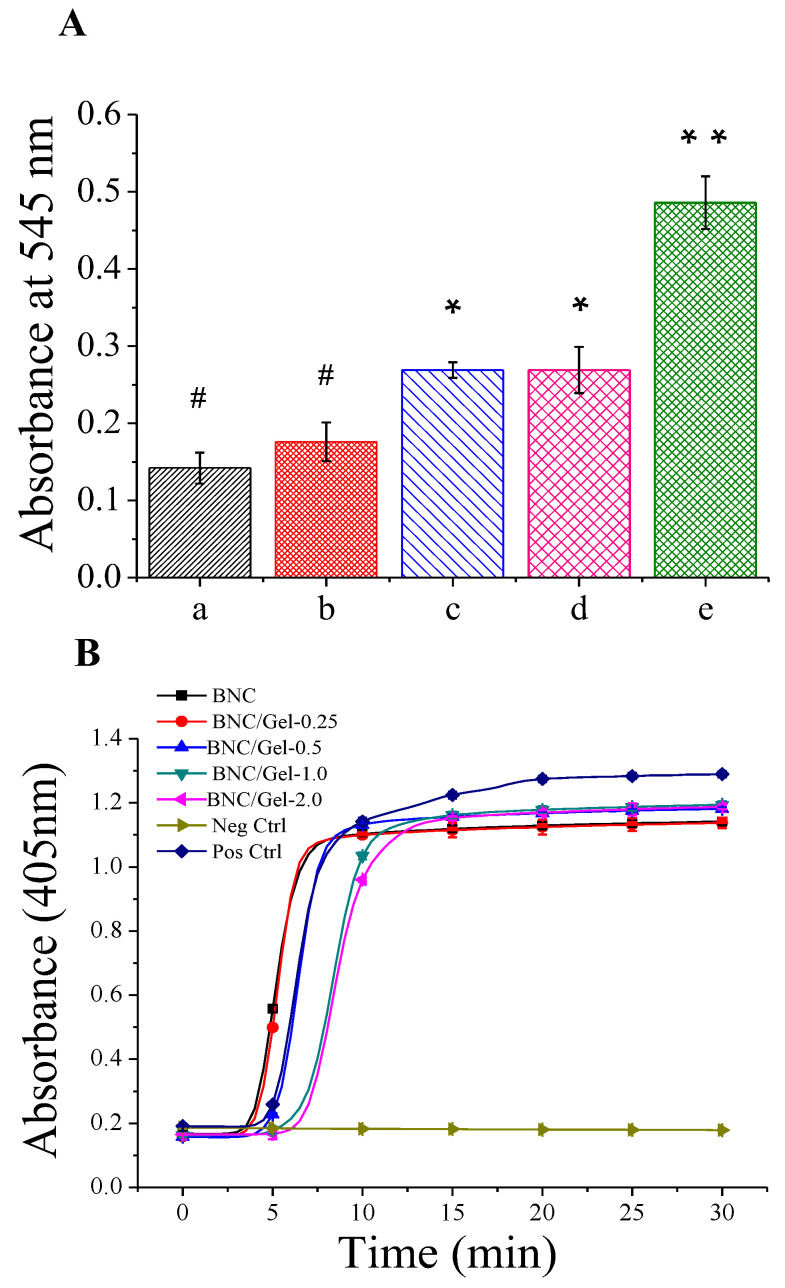
(**A**) Hemolytic rate analysis of BNC tube (a), BNC/Gel-0.25 (b), BNC/Gel-0.5 tube (c), BNC/Gel-1.0 tube (d) and BNC/Gel-2.0 tube (e). (**B**) Plasma recalcification dynamics process of BNC and BNC/Gel tubes along with controls. (** *p* < 0.01, * 0.01 < *p* < 0.05, # *p* > 0.05).

The hemolytic ratio reflects the degree of rupture of red blood cells, which is an important indicator of blood compatibility. Figure 4A shows that the hemolytic rates of pristine BNC and composite tubes are smaller than 1.0%, which indicates that they are non-hemolytic according to ISO10993-4:2002. The hemolytic ratio of BNC/Gel-0.25 had no significant difference from that of the BNC tube. However, with the increase in the amount of fish gelatin in the composite tubes, the hemolysis ratio increased slightly. The reason that fish gelatin promotes hemolysis over BNC may be that gelatin is a type of protein, whose structure is similar to the biological tissue structure.

(2) Plasma recalcification assay

The plasma recalcification dynamics process is a reproduction of the endogenous clotting process via the addition of calcium to the calcium-depleted plasma. Due to the accumulation of clotting by-products, the plasma becomes more turbid and the absorbance value becomes higher, until the plasma is completely coagulated and reaches the maximum absorbance. The plasma recalcification time is the measured time required to reach half of the maximum absorbance value in this test. The longer the time required, the less likely the material is to cause clots [36,37]. Figure 4B shows the coagulation times of BNC and BNC/Gel tubes. As shown in the figure, the curves representing the plasma recalcification kinetics process of the BNC/Gel-1.0 and BNC/Gel-2.0 tubes were more gentle, and they required more time to reach the maximum absorbance. The plasma recalcification times of BNC/Gel-2.0 and BNC/Gel-1.0 tubes (around 9 min) were significantly longer than that of the pristine BNC tube (around 5 min), which indicated that the BNC/Gel-1.0 and BNC/Gel-2.0 tubes performed the slowest stimulation of the clotting process. The coagulation time of the BNC/Gel-0.5 tube was approximately 8 min, which was longer than that of the pristine BNC tube and shorter than that of the BNC/Gel-1.0 and BNC/Gel-2.0 tubes. Moreover, the plasma recalcification times of the BNC and BNC/Gel-0.25 tubes had no significant difference, which means that the low content of fish-derived gelatin (0.25%) in the composite had no significant effect on the plasma recalcification time. The results indicated that the plasma recalcification time increases as the composite’s gelatin concentration increases within a certain concentration range. At present, the prevention of thrombosis is one of the key issues that needs to be solved in regard to small-caliber artificial blood vessels. This is of great significance for any material used as an artificial blood vessel.

(3) Whole blood clotting time

**Figure 5:**
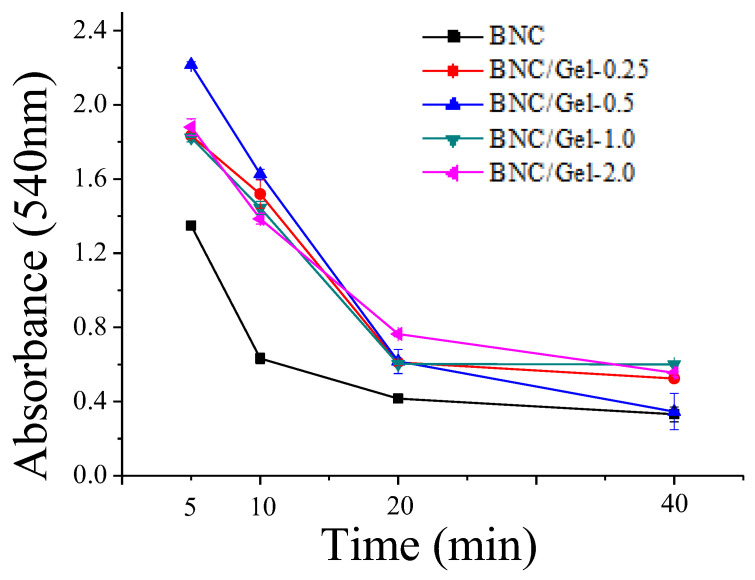
Whole blood clotting assay on the composite tubes.

The whole blood clotting assay reflects the influence of a material on whole blood coagulation [6]. Clotting occurs when whole blood is in contact with a biomaterial; the red blood cells retained in the clots cannot burst after the addition of ultra-pure water, which causes reduced hemoglobin release, and a low absorbance value is observed. A promising material with good anticoagulation activity will therefore maintain a higher absorbance value over time [14]. Figure 5 shows the whole blood clotting dynamic assay results of BNC and BNC/Gel tubes. As shown in the figure, the sample incubated with the BNC tube presented obviously lower absorbance than those with composite tubes at each measurement time point, which indicated that the introduction of fish gelatin was beneficial to improve the whole blood anticoagulant performance. Unlike the above two blood assays (hemolytic ratio and plasma recalcification assay), where no significant difference in performance could be found between BNC and BNC/Gel-0.25 tubes, the whole blood coagulation of the BNC/Gel-0.25 tube was significantly slower than that of the BNC tube, suggesting that a small amount of fish gelatin could improve the anticoagulation performance of whole blood. The results were consistent with a previous report, where gelatin attenuated platelet aggregation, slowed platelet-based thrombosis, inhibited the platelet adhesion function and significantly affected the plasma von Willebrand factor (vWF) function [38].

It has been reported that hemolysis is associated with hypercoagulable states, which potently activate platelets in vitro and in vivo [39]. In this study, gelatin improved the anticoagulation properties of BNC and also improved the hemolysis rate, which seems inconsistent with the conclusion reported. The hemolysis rates in this study were all much lower than 1%; therefore, the low hemolysis rate would have a negligible effect on the promotion of coagulation, and other factors affecting the coagulation effect could be considered. From the evaluation of the blood compatibility of composite tubes, BNC/Gel-1.0 possessed a lower hemolysis rate but a higher plasma recalcification time and whole blood coagulation time, indicating that it is the most suitable anticoagulant material.

### 3.4. In Vitro Cytocompatibility Assay

(1) The growth of HUVECs

**Figure 6:**
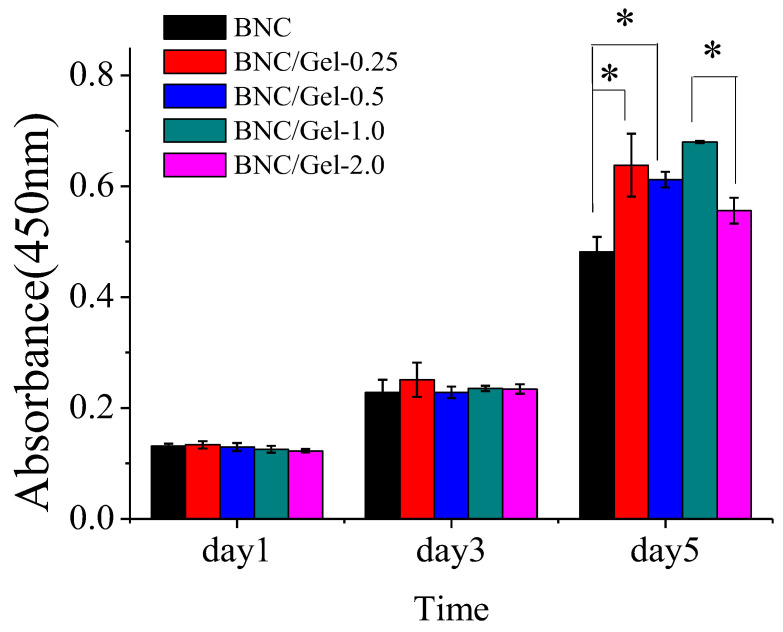
Proliferation of HUVECs on the surfaces of BNC tube, BNC/Gel-0.25, BNC/Gel-0.5 tube, BNC/Gel-1.0 tube and BNC/Gel-2.0 tube. (* *p* < 0.05).

**Figure 7:**
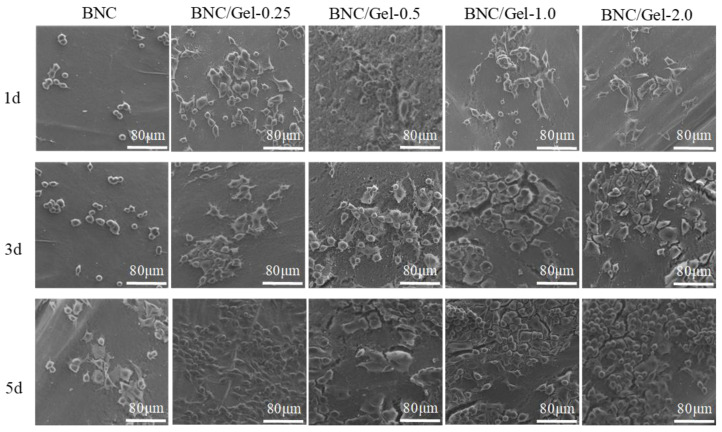
SEM images of HUVECs grown on the surfaces of samples.

In order to test the cytocompatibility of these materials, HUVECs were seeded on the BNC and BNC/Gel tubes. As shown in Figure 6, HUVECs cultured on all samples continually proliferated during 5-day incubation, indicating that all materials supported HUVECs’ proliferation without toxic effects. There was no significant difference in the number of cells adhered on all samples at the same time on day 1 and day 3. However, on day 5, the number of cells on the BNC/Gel composite tubes was significantly higher than that on the pure BNC tube, but it was not the case that more gelatin composited always provided a better proliferation effect. The number of cells on the BNC/Gel-1.0 tube was obviously the largest on day 5. The cells’ adhesion on the different tubes was detected by using SEM (Figure 7). We observed the proliferation and morphology of HUVECs cultured on the inner surfaces of BNC and BNC/Gel composite tubes. The numbers of cells on the BNC/Gel composite tubes were significantly higher than that on the BNC tube, and the cells were spread better, which may be due to the excellent biocompatibility of fish gelatin, benefiting cell adhesion, proliferation and spreading. Due to the growth of HUVECs, the BNC/Gel-1.0 tube was most beneficial for the adhesion and proliferation of HUVECs.

(2) The growth of HSMCs

**Figure 8:**
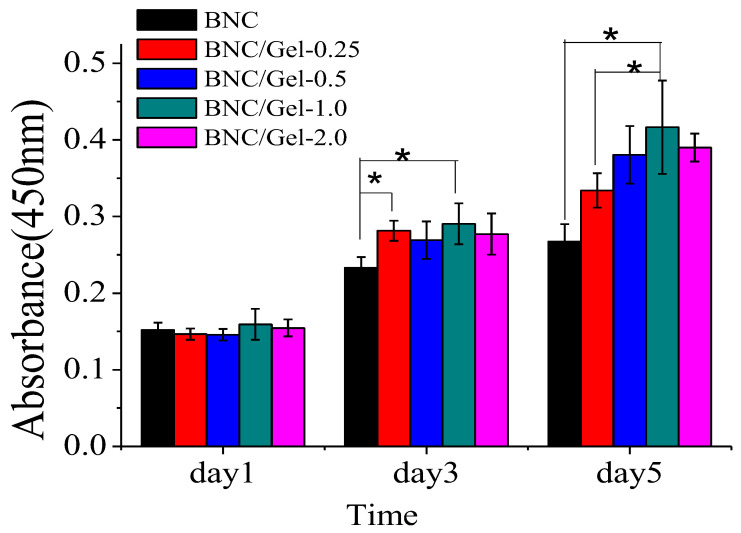
Proliferation of HSMCs on the surfaces of BNC, BNC/Gel-0.25, BNC/Gel-0.5, BNC/Gel-1.0 and BNC/Gel-2.0 tubes (* *p* < 0.05).

**Figure 9:**
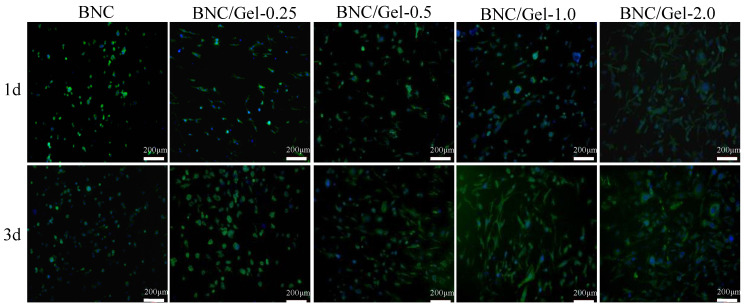
The fluorescence images of HSMCs grown on the surfaces of samples.

From the CCK-8 test results, we can observe the difference in the number of cells grown on different samples at different time points (day 1, 3, 5) (Figure 8). There was no significant difference in the number of cells adhered to various materials on the first day. After 5 days of cultivation, the numbers of cells on BNC/Gel tubes were significantly higher than that on the pure BNC tube. Moreover, the largest number of cells was observed on the BNC/Gel-1.0 tube. The cell morphology of HSMCs grown on the lumen surfaces of tubes was investigated by using fluorescent staining and observed using fluorescence microscopy (Figure 9). On day 1, the morphology of HSMCs on the pure BNC tube was different from that on BNC/Gel tubes. The cells on the pure BNC tube appeared to present oval shapes, but they showed fusiform shapes on the BNC/Gel tubes. After 3 days of cultivation, cells on the composite tubes grew densely and the cells had a specific fusiform morphology. The fluorescence images showed that the cells on the BNC/Gel-1.0 tube had a well-defined cytoskeleton organization and a larger cell density.

The reasons for this phenomenon could be based on the following aspects. (i) The adhesion of cells is related to the cells’ surface integrin receptor protein and the extracellular matrix (ECM) secreted by cells [16]. Gelatin is a degradation product of collagen and has the property of promoting cells’ adhesion and proliferation. (ii) High concentrations of fish gelatin might produce cytotoxicity or change the physical structure of the inner surface of the tube wall, which is detrimental to cells’ adhesion, proliferation and differentiation.

## 4. Conclusions

Pristine BNC and BNC/Gel composite tubes (compounded with different concentrations of fish gelatin) were prepared, and their biochemical properties, mechanical properties, hemocompatibility and cytocompatibility were evaluated by comparison. Results revealed that BNC/Gel tubes had improved mechanical properties to some extent, such as in terms of tensile strength and Young’s modulus. The hemolysis ratios of all samples were less than 1.0%, satisfying the requirements of implantable biomedical devices. The introduction of gelatin did not reduce the hemolysis ratio, but it significantly prolonged the plasma recalcification time and the whole blood clotting time of the pristine BNC tube. The evaluation of the blood compatibility of the tubes showed that the effect of the gelatin amount on each anticoagulant index was different. However, in the cytocompatibility experiments, it was not the case that a greater amount of fish gelatin was more conducive to HUVECs’ and HSMCs’ proliferation, and there is an optimal amount of fish gelatin. The BNC/Gel-1.0 tube might be more conducive to cell proliferation and spreading. The BNC/Gel-1.0 tube, with better compliance, demonstrated great potential for small-caliber artificial blood vessels and will be studied further as an artificial blood vessel material via in vivo animal experiments.

## Figures and Tables

**Table 1:** Characterization of BNC/Gel composite tubes.

Characteristics	BNC	BNC/Gel-0.25	BNC/Gel-0.5	BNC/Gel-1.0	BNC/Gel-2.0
Fish gelatin content (*w/w*%)	0	0.15 ± 0.04	0.16 ± 0.05	0.18 ± 0.01	0.23 ± 0.039
Permeability (mL·cm^−2^·min^−1^)	4.2 ± 0.4	4.2 ± 0.3	4.5 ± 0.4	4.6 ± 0.4	5.2 ± 0.3
Burst pressure (mmHg)/(MPa)	660.1 ± 65.0/ (0.088 ± 0.008)	592.6 ± 64.0/ (0.079 ± 0.009)	517.5 ± 54.5/ (0.069 ± 0.007)	465.0 ± 53.9/ (0.062 ± 0.007)	425.0 ± 46.3/ (0.057 ± 0.006)
Tensile strength (MPa)	0.51 ± 0.01	0.55 ± 0.09	0.62 ± 0.06	0.72 ± 0.05	0.85 ± 0.02
Young’s modulus (MPa)	0.98 ± 0.10	1.01 ± 0.15	1.09 ± 0.22	1.07 ± 0.18	1.19 ± 0.11
Elongation at break (%)	38.6 ± 0.5	36.6 ± 0.3	38.8 ± 0.5	41.6 ± 0.5	43.9 ± 0.3

## Data Availability

All data generated or analyzed during this study are included in this article.
